# Perceived Stress During the COVID-19-Related Confinement in Cyprus

**DOI:** 10.3389/fpubh.2021.673411

**Published:** 2021-06-02

**Authors:** Maria Kyprianidou, Costas A. Christophi, Konstantinos Giannakou

**Affiliations:** ^1^Cyprus International Institute for Environmental and Public Health, Cyprus University of Technology, Limassol, Cyprus; ^2^Department of Health Sciences, School of Sciences, European University Cyprus, Nicosia, Cyprus

**Keywords:** mental health, perceived stress, COVID-19, quarantine, public mental health, Cyprus

## Abstract

The COVID-19 pandemic is a serious global health emergency that could potentially have a significant impact on both somatic as well as psychological level. The aim of this study was to assess the prevalence of perceived stress in the general adult population of Cyprus during the first COVID-19 lockdown period. This was an internet-based cross-sectional study conducted between 6 April and 20 June 2020, one to two and a half months after the introduction of and the first mandatory lockdown on its entire territory imposed by the government of the Republic of Cyprus on 24 March 2020. Data collection was done using a self-administered questionnaire that included information about socioeconomic and demographic characteristics, physical activity, smoking habits, and stress level. A total of 1,485 adults participated in the study. The median perceived stress score was 10 (q1 = 6, q3 = 15). Linear regression models showed that having a medium monthly income (€501-1,500) and being a current smoker was positively associated with the perceived stress score, while being male and physically active was negatively associated with the perceived stress score (all *p* <0.05). People with medium average salary and current smokers were at a higher risk for perceived stress. Psychological interventions and/or psychological services provided in certain vulnerable groups would be beneficial in future lockdowns due to either COVID-19 or a new pandemic.

## Introduction

The outbreak of the coronavirus disease COVID-19, caused by SARS-CoV-2, in China in early December 2019 was rapidly transmitted globally and developed into a pandemic by March 2020 (1–3). Under this public health emergency, most countries implemented unprecedented policies and strategies to contain the spread of the disease, ultimately resulting in around a third of the global population being subjected to COVID-19 related lockdowns (4). Lockdown measures included travel restrictions and the closure of educational institutions, retail, and industries, except for essential services (e.g., hospitals, police, groceries, etc.). Citizens were forced to quarantine themselves at home and advised to maintain social distance whilst they were allowed to leave their home only for essentials (e.g., food markets, pharmacies, and healthcare).

While physical distancing and quarantine orders can be effective measures to tackle the rapid spread of the COVID-19 virus, they can also have serious effects on mental health. The quarantine enforced due to the COVID-19 pandemic can potentially contribute to the development of mental health problems (such as anxiety, stress, fear, trauma, risk of depression, post-traumatic stress disorder, etc.) (5–7) due to many reasons including among others the fear of infection, psychosocial stressors (e.g., financial strain, social isolation, food insecurity, unemployment, etc.), the lack of medical treatment, or even because of stress caused by the uncontrollable and unpredictable nature of COVID-19 (5, 8, 9). Emerging literature indicates that various psychological distress reactions are occurring in response to COVID-19 lockdown measures. For instance, in countries such as China, Italy, Greece, and Spain, where strict quarantine measures were implemented, recent internet-based cross-sectional studies demonstrated a high frequency of depressive, stress, and anxiety symptoms (10–15). At the same time, small-scale studies conducted in Cyprus during the first lockdown period indicated a relatively high prevalence of psychological symptoms (e.g., anxiety, insomnia, somatic, and depressive symptoms) (16, 17). However, to the best of our knowledge, the distress experienced by people during COVID-19 pandemic and related lockdown measures has not been explored so far in Cyprus. Therefore, the aim of this study was to assess the prevalence of perceived stress during the first COVID-19 lockdown period on the general adult population of Cyprus and identify factors associated perceived stress. Ultimately, this could help public health authorities to plan effective mental health management strategies, for this and future epidemics.

## Materials and Methods

### Study Design, Participants, and Procedure

This was a cross-sectional study. The referent population included Cypriot Greek-speaking men and women aged 18 years and older, residents of the five government-controlled districts of the Republic of Cyprus [(Nicosia, 54% of the total Cypriot population, Limassol (23%), Larnaca (14%), Paphos (6%), and Ammochostos (3%)] during the first wave of the COVID-19 pandemic.

The data were collected using a standardized online questionnaire. The questionnaire was administered using Google Forms between 6 April 2020 and 20 June 2020 (one to two and a half months after the initiation of the social confinement measures and the first mandatory lockdown on its entire territory imposed by the government of the Republic of Cyprus on 24 March 2020). The online questionnaire was dispersed using social media apps (i.e., Facebook), instant messaging apps (i.e., WhatsApp, Viber, and Messenger), social networking (i.e., LinkedIn), and emails through snowball sampling. The dissemination of the questionnaire was initiated after the approval of the study by the Cyprus National Bioethics Committee (CNBC).

### Instruments and Variables

The online self-administered questionnaire contained questions on demographic characteristics (i.e., age in years, gender, marital and educational status, and annual income), lifestyle habits such as physical activity, smoking status, and perceived stress.

Marital status was classified as unmarried, married/engaged, or separated/divorced/widowed. Education level was recorded into three categories (primary education, secondary education, and higher education) while salary status was assessed using the personal monthly salary and categorized as low income (≤ €500), middle income (€501-1,500), and high income (>€1,500). Smoking status was recorded as current smoker, past smoker, or never smoker, while for the assessment of physical activity, participants had to report if they were physically active or not, the sport/physical activity that they mainly did (e.g., walking, inhouse exercise etc.), and the duration of it in hours per week. Body mass index (BMI) was calculated as weight (in kilograms) divided by standing height (in meters) squared and then the BMI groups were defined as obese if BMI > 29.9 kg/m^2^, overweight as BMI 25-29.9 kg/m^2^, normal as BMI 18.5-24.9 kg/m^2^, and underweight as BMI <18.5 kg/m^2^, according to the WHO classification. More details regarding dietary assessment and adherence to the Mediterranean diet during the first COVID-19 lockdown period among the same population is presented elsewhere (18).

To assess the levels of perceived stress we used the Greek version of Perceived Stress Scale-14 (PSS-14) (19), which is a measure of the degree to which situations in one's life are appraised as stressful and consists of seven negative and seven positive items. Each question had one possible answer rated on a five-point scale (never, almost never, sometimes, fairly often, very often). The positive items evaluate the ability to cope with perceived stressors, whereas the negative ones assess the lack of control and negative emotions and reactions. The highest possible score is 56 and scores <11, 12-15, and >16 are considered as evidence of low, moderate, and high perceived stress, respectively.

### Statistical Analysis

Continuous variables with normal distribution are presented as mean ± standard deviation (SD) while continuous variables with not normal distribution as median (q_1_, q_3_). The distributions of continuous variables were assessed for normality using the Shapiro-Wilk test. Categorical variables (i.e., sex, marital, educational, and salary status, physical activity, smoking status, and BMI categories) are presented as frequencies (%). Normally distributed variables were compared among the perceived stress categories using the ANOVA technique while non-normally distributed variables were compared using the non-parametric Kruskal-Wallis test. The distributions of categorical characteristics in the different perceived stress groups were compared using the Pearson's chi-squared test of independence.

Multivariate linear regression models were used to evaluate the association of demographics, socioeconomic, and lifestyle characteristics with the perceived stress score in the Cypriot population during the COVID-19 confinement. The results were reported as beta coefficients (SE). Hierarchical ordinal logistic regression models were also used on the perceived stress score categories (low, moderate, and high) with demographics (i.e., age, gender), socioeconomics (i.e., marital, educational, and salary status), and lifestyle characteristics (i.e., physical activity level, smoking status) as covariates. First, we adjusted for age and sex, then added socioeconomic characteristics, and, finally, lifestyle habits were added. The fit of the models was assessed using the Hosmer-Lemeshow goodness of fit test. The results are presented as Odds Ratios (OR) with the corresponding 95% Confidence Intervals (CI). All statistical hypotheses were two-sided with statistical significance level set at α = 0.05. Statistical analysis was conducted using STATA 14.0 statistical software (Stata Corp, College Station, TX, USA).

### Ethics Approval

This study was conducted according to the Declaration of Helsinki guidelines and all procedures involving research study participants were approved by the Cyprus National Bioethics Committee (EEBK EΠ 2020.01.57). The application submitted to the CNBC along with the relevant questionnaire outlined the study objectives and outcomes, the data collection process and data management, the use of the data, and the expected benefits. Participation was anonymous and all the participants were informed about the aim and objectives of the study before participating.

## Results

### Participants' Characteristics

A total of 1,485 Cypriot adults participated in the study. The median age of all participants was 34 (q1 = 27, q3 = 43) years old (Table 1). Among the participants, 60% were females, 54% were residents of Nicosia, most of the participants had completed a higher education (82%), 52% were married, and 45% were categorized as having a high monthly salary (>€1,500). Most of the participants never smoked (65%) and were physically active (66%).

**Table 1:** Baseline characteristics and perceived stress during the first COVID-19 lockdown period.

**Characteristics**	**Overall (*N* = 1485)**	**Low(*N* = 850)**	**Moderate (*N* = 268)**	**High(*N* = 367)**	***P*-value**
**Perceived stress score^a^**	10 (6, 15)	7 (4, 9)	13 (12.5, 15)	20 (17, 24)	** <0.01**
**Age**	34 (27, 43)	35 (28, 45)	34 (27, 43)	32 (25, 40)	** <0.01**
**Age group**					** <0.01**
18-24	280 (18.9)	142 (16.7)	51 (19.1)	87 (23.7)	
25-44	862 (58.1)	484 (56.9)	159 (59.3)	219 (59.7)	
45+	343 (23.1)	224 (26.4)	58 (21.6)	61 (16.6)	
**Gender**					** <0.01**
Female	833 (60.0)	456 (54.2)	163 (60.8)	264 (73.1)	
Male	588 (40.0)	386 (45.8)	105 (39.2)	97 (26.9)	
**Geographical area**					
Nicosia	799 (54.1)	471 (55.9)	153 (57.3)	175 (47.8)	**0.02**
Limassol	341 (23.1)	187 (22.2)	55 (20.6)	99 (27.0)	
Larnaca	202 (13.7)	113 (13.4)	43 (16.1)	46 (12.6)	
Paphos	89 (6.0)	46 (5.5)	13 (4.9)	30 (8.2)	
Ammochostos	45 (3.1)	26 (3.0)	3 (1.1)	16 (4.4)	
**Residency**					0.47
Urban	1,232 (85.5)	702 (83.1)	228 (86.0)	302 (82.7)	
Rural	243 (16.5)	143 (16.9)	37 (14.0)	63 (17.3)	
**Marital status**					0.07
Married	764 (51.9)	449 (53.4)	139 (52.1)	176 (48.5)	
Unmarried	623 (42.4)	343 (40.8)	107 (40.1)	173 (47.7)	
Divorced/Widowed	84 (5.7)	49 (5.8)	21 (7.8)	14 (3.8)	
**Educational status**					0.43
Primary education	4 (0.3)	4 (0.5)	0 (0.0)	0 (0.0)	
Secondary education	258 (17.4)	141 (16.6)	50 (18.7)	67 (18.3)	
Higher education	1,221 (82.3)	704 (82.9)	218 (81.3)	299 (81.7)	
**Salary**					** <0.01**
Low	310 (20.9)	157 (18.6)	54 (20.2)	99 (27.0)	
Middle	509 (34.4)	284 (33.6)	92 (34.3)	133 (36.2)	
High	662 (44.7)	405 (47.7)	122 (45.5)	135 (36.8)	
**Physically active**					** <0.01**
Yes	975 (65.9)	260 (30.7)	104 (38.8)	141 (38.6)	
No	505 (34.1)	587 (69.3)	164 (61.2)	224 (61.4)	
**Smoking**					** <0.01**
Non-smoker	965 (65.1)	542 (64.0)	179 (66.8)	244 (66.5)	
Past smoker	191 (12.9)	172 (20.3)	61 (22.8)	93 (25.3)	
Current smoker	326 (22.0)	133 (15.7)	28 (10.4)	30 (8.2)	
**BMI group**					** <0.01**
Underweight	72 (4.9)	35 (4.2)	18 (6.7)	19 (5.2)	
Normal	743 (50.5)	406 (48.3)	128 (47.8)	209 (57.6)	
Overweight	421 (28.6)	266 (31.7)	79 (29.5)	76 (20.9)	
Obese	235 (16.0)	133 (15.8)	43 (16.0)	59 (16.3)	

a *median (Q1, Q3)*.

*Bold indicates statistically significant at a P <0.05*.

### Perceived Stress Scale—PSS-14

The median perceived stress score of the participants was 10 (q1 = 6, q3 = 15) with the maximum score being 48. We observed differences among the three age groups, between males and females, in the five geographical areas of Cyprus, among the salary categories, physical activity and smoking status levels, and BMI categories (Supplementary Figure 1).

Our results show that in the high perceived stress group, there were more individuals aged 18-24 years old than >45 years old (24 vs. 17%) compared to the other two perceived stress groups (*p* <0.01) (Table 1). Furthermore, in the high group there were more females than males (73 vs. 27%) in comparison to the low group (54 vs. 46%). Although, most of the residents in each of the five geographical areas of Cyprus were classified in the low group, we noticed that 29, 34, and 36% of the residents of Limassol, Paphos, and Ammochostos, respectively, were in the high group (*p* = 0.02). As regards to the socioeconomic characteristics, we found statistically significant associations only among the salary categories and stress groups. In particular, more individuals (48%) with high salary were in the low group compared to the corresponding percentages of the other two (*p* <0.01). In addition, most of the participants were physically inactive in all the stress categories and the largest percentage of physically inactive participants was identified in low stress group (69%) (*p* <0.01). Moreover, in the three categories of stress, the lowest percentages were reported for current smokers among the three categories of smoking. More specifically, we reported 16, 10, and 8% current smokers in low, moderate, and high stress group, respectively (*p* <0.01). Lastly, we found statistically significant differences among BMI and stress categories (*p* <0.01) (Table 1).

Multivariate linear regression models indicated that being a current smoker was positively associated with the perceived stress score (Table 2). In addition, being male, having a middle or a high average salary, and being physically active was negatively associated with the perceived stress score. Specifically, being a current smoker increases the score of the perceived stress scale by 1.41 units (95% CI: 0.46, 2.35) (*p* < 0.01) while having middle or having high monthly income, being male, or being physically active decreases the score by 1.38 (95% CI: −2.56, −0.19), 1.35 (95% CI: −2.70, −0.01), 1.99 (95% CI: −2.80, −1.17), and 1.16 units (95% CI: −1.97, −0.35), respectively (all *p* < 0.05) (Table 2).

**Table 2:** Multivariate linear regression for the factors affecting level of the perceived stress score in Cypriot population during the first COVID-19 lockdown period.

**Characteristics**	**β-coefficient (95% CI)**
Age	−0.03 (−0.07, 0.02)
Male vs. female	**−1.99 (−2.80**, **−1.17)**
**Marital status**	
Married	Ref
Unmarried	−0.04 (−1.06, 0.97)
Divorced/widowed	−0.83 (−2.52, 0.86)
Higher vs. Secondary education	−0.01 (−1.13, 1.12)
**Salary**	
Low	Ref
Middle	**−1.38 (−2.56**, **−0.19)**
High	**−1.35 (−2.70**, **−0.01)**
Physically active vs. inactive	**−1.16 (−1.97**, **−0.35)**
**Smoking**	
Non-smoker	Ref
Current smoker	**1.41 (0.46, 2.35)**
Past smoker	−1.10 (−2.29, 0.07)

*Bold indicates statistically significant at a P <0.05*.

The ordinal logistic regression analysis did not reveal any statistically significant associations of age, gender (Table 3, Model 1), or socioeconomic factors (Table 3, Model 2) with the odds of being in the moderate vs. low group. When we further adjusted for lifestyle factors, we found that physical activity participants had a lower risk of having moderate stress compared to physically inactive participants (Table 3—Model 3; OR = 0.73; 95% CI: 0.55, 0.98).

**Table 3:** Hierarchical ordinal logistic regression (moderate vs. low) to evaluate the association of demographics (i.e., age, gender), socioeconomic factors (i.e., marital, educational, and salary status), and lifestyle factors (i.e., physically activity level and smoking status) on the level of perceived stress (defined through PSS-14).

**Characteristics**	**Moderate Perceived stress Odds Ratio (95% CI)**		
	**Model 1: Adjusted for age and gender**	**Model 2: Adjusted for socio-economic factors**	**Model 3: Adjusted for lifestyle factors**
Age	0.99 (0.98, 1.01)	0.99 (0.97, 1.00)	0.99 (0.97, 1.00)
Male vs. female	0.76 (0.58, 1.01)	0.78 (0.58, 1.04)	0.82 (0.61, 1.11)
**Marital status**			
Married		Ref	Ref
Unmarried		0.84 (0.56, 1.22)	0.86 (0.59, 1.26)
Divorced/widowed		1.47 (0.84, 2.57)	1.44 (0.82, 2.53)
Higher vs. Secondary education		0.82 (0.54, 1.23)	
**Salary**			
Low		Ref	Ref
Middle		1.09 (0.70, 1.70)	1.09 (0.69, 1.70)
High		1.13 (0.68, 1.88)	1.16 (0.70, 1.93)
Physically active vs. inactive			**0.73 (0.55, 0.98)**
**Smoking**			
Non-smoker			Ref
Current smoker			1.07 (0.75, 1.52)
Past smoker			0.74 (0.47, 1.16)

*Bold indicates statistically significant at a P < 0.05*.

Gender was a statistically significant predictor of the probability of being in the high group compared to low group (Table 4—Model 1; OR = 0.45; 95% CI: 0.35, 0.60), and the association remained statistically significant even after adjusting for socio-economic factors (Table 4—Model 2; OR = 0.46; 95% CI: 0.35, 0.61) and even after further adjustment for lifestyle factors (Table 4—Model 3; OR = 0.48; 95% CI: 0.36, 0.64). After the addition of socio-economic factors, we did not identify any statistically significant associations whereas when we added lifestyle factors smoking status was found to be statistically significant with current smokers having 1.37 times higher risk of being in the high Perceived stress score group compared to non-smokers (*p* < 0.01) (Table 4).

**Table 4:** Hierarchical ordinal logistic regression (high vs. low) to evaluate the association of demographics (i.e., age, gender), socioeconomic factors (i.e., marital, educational and salary status) and lifestyle factors (i.e., physically activity level and smoking status) on the level of perceived stress (defined through PSS-14).

**Characteristics**	**High Perceived stress Odds Ratio (95% C.I)**		
	**Model 1: Adjusted for age and gender**	**Model 2: Adjusted for socio-economic factors**	**Model 3: Adjusted for lifestyle factors**
Age	0.98 (0.97, 0.99)	0.99 (0.98, 1.01)	0.99 (0.98, 1.01)
Male vs. female	**0.45 (0.35, 0.60)**	**0.46 (0.35, 0.61)**	**0.48 (0.36, 0.64)**
**Marital status**			
Married		Ref	Ref
Unmarried		1.00 (0.71, 1.39)	0.99 (0.70, 1.40)
Divorced/widowed		0.74 (0.39, 1.40)	0.70 (0.37, 1.33)
Higher vs. Secondary education		1.02 (0.70, 1.49)	
**Salary**			
Low		Ref	Ref
Middle		0.78 (0.53. 1.13)	0.73 (0.50, 1.07)
High		0.67 (0.43, 1.04)	0.67 (0.43, 1.04)
Physically active vs. inactive			0.78 (0.59, 1.02)
**Smoking**			
Non-smoker		Ref	Ref
Current smoker			**1.37 (1.01, 1.87)**
Past smoker			0.74 (0.47, 1.15)

*Bold indicates statistically significant at a P < 0.05*.

## Discussion

To the best of our knowledge, our study is the first to report the prevalence of perceived stress in the general population of Cyprus during the first COVID-19 lockdown period. The median Perceived stress score of the participants was 10 (q1 = 6, q3 = 15), which indicated a low stress level in the general population of Cyprus during the first COVID-19 lockdown period. Perceived stress was more likely to occur in individuals that had a middle average salary and were current smokers, while being male and physically active was negatively associated with perceived stress.

To avoid the further spread of the COVID-19 outbreak, the government of the Republic of Cyprus took early in the pandemic social distancing measures and a mandatory national lockdown for 8 weeks. On one hand, it may be assumed that the COVID-19 home confinement for individuals with high levels of socioeconomic security, the avoidance of commuting, changes in work activities, and increased time with family potentially could have reduced stress. On the other hand, individuals could become more anxious, stressed, or agitated due to the physical distancing and self-isolation measures as well as about the possibility of COVID-19 infection. In fact, despite the lockdown situation, perceived stress was found to be in low and moderate ranges in our study. Our findings concur with other web-based cross-sectional surveys conducted during COVID-19 lockdown. Specifically, a study in India, in which the majority of the participants were females with mean age 30 years old, similar to our study participants, found that the levels of stress were mild (20), while another epidemiological study conducted in China reported only a small percentage (8.1%) of participants to have moderate to severe stress levels (7). Also, in a study that assessed stress in nearly 10,000 participants across 78 countries including Cyprus, stress was found to be moderate for most people (55.9%), and 11% reported the highest levels of stress (21). Of interest, in our study we have also observed that almost a quarter of the population had a high perceived stress (23%) something that is consistent with similar studies in other European countries (14, 22). In a similar study that assessed stress using the Perceived Stress Scale (PSS-10) and included participants from 41 countries [i.e., Philippines (43%), Spain (30%), and Colombia (11%)] reported a higher score of perceived stress compared to our study (17 vs. 10) (23).

It is likely that the lockdown measures due to the COVID-19 pandemic did not affect all groups equally, with individuals in some demographic subgroups being affected in various degrees, with regards to their mental distress, during the pandemic. Our findings show significant differences in the level of perceived stress, between males and females and among the people who had a middle salary compared to those with a low salary. It is true that there is the perception that females have higher stress than males since, the Hypothalamo-Pituitary-Adrenal (HPA) plays an important role in the neuroendocrine response to stress, with females typically presenting higher responses compared to males (24). Correspondingly, during the period of COVID-19 outbreak, we reported higher score of perceived stress in women compared to men, an observation which agrees with other epidemiological studies (7, 23, 25). Concerning income status, we observed that people with middle income had a higher risk of reporting higher score in perceived stress scale in comparison with those with low income. It is likely that lockdown measures implemented due to COVID-19 pandemic have caused an acute financial strain, taking into account that some people may have lost their jobs, seen their income plummet, or been furloughed (26). In our study, most of the participants who classified their salary status as low income were mainly unemployed (90%), of which 76% were between 18 and 24 years old, 16% were between 25 and 44 years old, and 8% were 45 years old and older, while the largest percentage among people with middle income was private employees (60%). Therefore, it is plausible that individuals who were unemployed before the COVID-19 lockdown, they did not face a new situation which would likely cause them stress, in contrast with those with middle income, among whom it is more likely that many of them lost their jobs or received a reduced salary because of the COVID-19 pandemic. Our findings also show significant differences in the level of perceived stress and geographical area. We have no concrete evidence as to why the percentages are higher in cities of Limassol, Paphos, and Ammochostos and there are no other published studies that we are aware of, that examine these specific geographical areas in relation to stress. Our speculation is that this may be due to individuals in those seaside cities previously having access to beach and opportunities to exercise outdoors, as part of their daily routines and therefore experienced higher levels of stress compared to individuals living in the interior of the country that did not previously incorporate such routines in their habits.

Regarding the participants' lifestyle characteristics, we observed that physically active people are less likely to have higher levels of perceived stress compared to inactive people, while current smokers were more likely to have a higher score of perceived stress scale compared to non-smokers. Although, a common belief is that smoking reduces stress, previous research suggests that smoking could be associated with altered functioning of the HPA axis and the Autonomic Nervous System (ANS) (27), that may generate or aggravate negative emotional states and propagate negative coping strategies leading to overall higher stress levels (28–30). With regards to physical activity, possible mechanism hypotheses suggest that it neutralizes the effects of psychological stressors on cardiac reactivity and dampens stressor-evoked increases in stress hormones and serotonin (31–33), whilst physical activity could also enhance mental health *via* changes in the structural and functional composition of the brain (34).

This study has several strengths. To the best of our knowledge, this is the first study examining the prevalence of perceived stress during the first COVID-19 lockdown period. In addition, our study has a relatively large sample size, both males and females (>18 years old) from different geographical areas of Cyprus which allows us to perform a robust analysis. There were also some limitations. Firstly, due to the cross-sectional design of the study, it is difficult to make causal inferences. Secondly, the study was limited to the COVID-19 outbreak and we used a convenient online survey that limits the sample representativeness and is prone to bias due to misreporting of self-report data. However, the use of a web-based survey is an alternative solution for data collection in periods of social distancing and our sample included participants from different geographical area and age groups. Thirdly, this study assessed perceived stress during the first mandatory lockdown imposed by the government of the Republic of Cyprus. Perceived stress might be modified in a prolonged confinement and in the various phases of the pandemic. Lastly, due to the sudden occurrence of the outbreak, we were unable to assess an individual's psychological conditions before the COVID-19 outbreak.

## Conclusion

This is the first study examining the impact of the first confinement imposed due to COVID-19 on the levels of perceived stress of the Cypriot adult population. Our findings show that the perceived stress score was at a low level in the general population of Cyprus during COVID-19 lockdown. In addition, perceived stress was positively associated with middle average salary individuals and being a current smoker, while being male and physically active was negatively associated with the perceived stress. Our results should be used as a starting point for further studies aiming to develop psychological interventions and/or psychological services. In addition, our results can provide evidence for comparison purposes in future studies of the long-term impact of these confinement measures given the extent of the COVID-19 pandemic. Public health initiatives are further urged to develop appropriate responses for certain vulnerable groups for future lockdowns or physical distancing due to either COVID-19 or a new pandemic.

## Data Availability Statement

The raw data supporting the conclusions of this article will be made available by the authors, without undue reservation.

## Ethics Statement

The studies involving human participants were reviewed and approved by the Cyprus National Bioethics Committee (EEBK EΠ 2020.01.57). Written informed consent for participation was not required for this study in accordance with the national legislation and the institutional requirements.

## Author Contributions

MK conceived and designed the web-survey, collected, and analyzed the data, and draft the original manuscript. CC contributed to the design of the study, the interpretation of the results, and in critically editing the original draft. KG supervised the study, conceived, and designed the web-survey, collected the data, draft the original manuscript, and interpret the results. All the authors take responsibility for all aspects of the reliability and freedom from bias of the data presented and their discussed interpretation. All authors read and approved the final manuscript.

## Conflict of Interest

The authors declare that the research was conducted in the absence of any commercial or financial relationships that could be construed as a potential conflict of interest.
