# Supplementary material for: Perceived Stress During the COVID-19-Related Confinement in Cyprus
Source: Front Public Health. 2021 Jun 2;9:673411. doi: 10.3389/fpubh.2021.673411 (PMC8206502; doi:10.3389/fpubh.2021.673411)

**Supplementary Figure 1:** Distribution of Perceived stress score overall and by age group, sex, geographical area, salary status, physical activity level and smoking status, and BMI categories


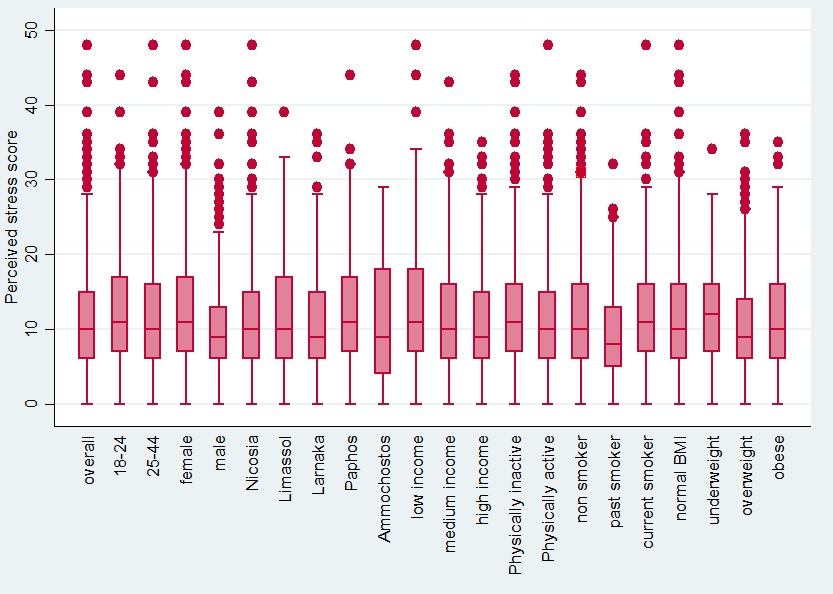

Supplement: Supplementary file 1 [file Table_1.DOCX]
